# Grip strength positively correlates with blood pressure in individuals with abnormal adiposity

**DOI:** 10.1038/s41371-023-00862-6

**Published:** 2023-09-09

**Authors:** Jedd Pratt, Nazareno Paolocci, Colin Boreham, Giuseppe De Vito

**Affiliations:** 1 Institute for Sport and Health, University College Dublin, Dublin, Ireland; 2 Department of Biomedical Sciences, CIR-Myo Myology Centre, Neuromuscular Physiology Laboratory, University of Padova, Padua, Italy; 3 Department of Biomedical Sciences, University of Padova, Padua, Italy; 4 Division of Cardiology, Johns Hopkins Medical Institutions, Baltimore, MD 21205 USA

**Keywords:** Risk factors, Hypertension

## Abstract

Although strong positive correlations exist between grip strength and cardiovascular health, the association between grip strength and blood pressure (BP) is less clear. In this regard, a more precise relationship between grip strength and BP may be revealed by considering adiposity. We examined the association between grip strength and BP in 9424 individuals aged 18–92 years, while controlling for or stratifying by body mass index (BMI) or body fat (BF)%. Grip strength, BP and BF% were determined using dynamometry, sphygmomanometry and dual-energy x-ray absorptiometry. Overall, those with elevated BP had greater grip strength than those with normal BP (39.17 kg vs 38.38 kg, *p* < 0.001); however, following stratification this was only observed in overweight or obese individuals (42.08 kg vs 41.10 kg, *p* = 0.003 and 41.34 kg vs 40.03 kg, *p* = 0.033), and those within the highest BF% tertile (37.95 kg vs 36.52 kg, *p* < 0.001). Overall, higher grip strength was associated with an increased odds for elevated BP (OR = 1.014, 95% CI = 1.004–1.024, *p* = 0.004); however, after stratification the increased odds was only observed in overweight or obese individuals (OR = 1.025, 95% CI = 1.010–1.039, *p* < 0.001 and OR = 1.018, 95% CI = 1.004–1.031, *p* = 0.010), and those within the highest BF% tertile (OR = 1.036, 95% CI = 1.022–1.051, *p* < 0.001). Individuals with low grip strength and high BF% had lower odds for elevated BP (OR = 0.514, 95% CI = 0.341–0.775, *p* = 0.002), whereas those with low grip strength and low BF% had higher odds for elevated BP (OR = 2.162, 95% CI = 1.026–4.555, *p* = 0.043). Our findings show that higher grip strength is related to higher BP in overweight or obese individuals, or those with a high BF%. Having a BMI < 25 kg/m^2^ or lower BF% may neutralise this association.

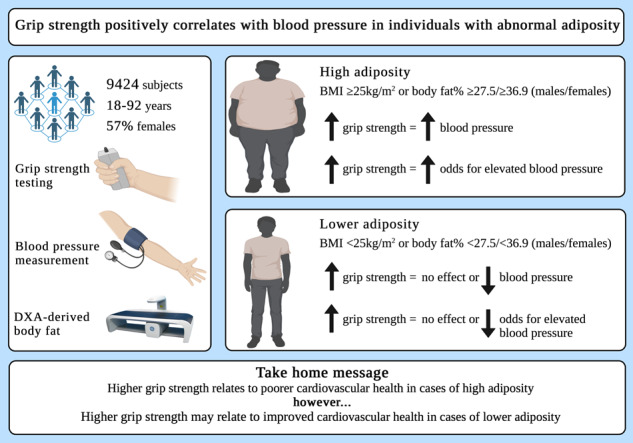

## Introduction

High blood pressure (BP) is a principal risk factor for cardiovascular disease and the leading contributor to disability-adjusted life years and mortality [1]. Globally, over 1.2 billion people have hypertension, defined as systolic blood pressure (SBP) of 140 mmHg or greater and diastolic blood pressure (DBP) of 90 mmHg or greater [2]. Several factors heighten individual risk of developing hypertension, broadly categorised into non-modifiable and modifiable. Non-modifiable factors include sex, age, family history, race, ethnicity, and genetics, and modifiable factors include an unhealthy diet, alcohol consumption, stress, smoking, physical inactivity, and obesity [3–6]. In particular, considerable evidence supports the benefits of physical activity, exercise, and physical fitness in ameliorating the prevalence and burden of hypertension [7, 8].

Grip strength is a well-established proxy for muscular fitness [9] and a strong predictor of clinical outcomes such as future disability, length of hospital stay, morbidity, and mortality [10–12]. Notably, a strong positive relationship exists between grip strength and cardiovascular health [13, 14]. With this in mind, and given that hypertension is a major risk factor for cardiovascular disease, it seems logical that grip strength would be negatively associated with BP. However, evidence supporting this hypothesis is conflicting. While some studies have reported an inverse relationship between grip strength, BP, and hypertension risk [15–17], several others have documented the opposite [18–20], or no association [21].

In light of this, we speculated that although grip strength has potential utility as a screening tool for CVD, focussing on its relationship with BP without considering other potentially mediating factors may not unveil its true screening potential. Accordingly, we turned our attention to another comorbidity, adiposity. In contrast to muscle fitness, high levels of adiposity consistently correlate with decreased cardiovascular health and increased BP [22, 23]. Furthermore, in keeping with our hypothesis, emerging data suggest that body mass index (BMI) may mediate the relationship between grip strength and BP, such that grip strength is positively associated with BP and hypertension risk in overweight or obese individuals (BMI ≥ 25 kg/m^2^) [18], but is negatively related to these parameters in normal weight individuals (BMI = 18.5–24.9 kg/m^2^) [16]. These findings suggest a positive relationship between muscular fitness and BP in those with an average body weight and that an increase in adiposity may reverse the effect direction.

However, several limitations of existing studies need to be addressed. Most importantly, all studies have employed BMI as the measure of adiposity, and are therefore limited by the inherent inaccuracies of BMI in determining adiposity in certain populations. For example, BMI does not incorporate a specific measure of adiposity, but rather classifies the level of adiposity based solely upon one’s total body mass and height. As a result, robust athletic individuals carrying large amounts of muscle mass can be inaccurately classified as overweight or obese, even if they have a healthy level of body fat (BF) [24]. In contrast, others may be classified as normal weight using BMI, yet may carry an unhealthy proportion of their body mass as adipose tissue if their muscle mass is low. With this in mind, there is a clear need to incorporate a more accurate measure of adiposity, such as that determined by dual energy x-ray absorptiometry (DXA). Additionally, modest sample sizes [20, 21], narrow age ranges [19, 21], and conflicting reports of sex-specific differences [15, 18] further hamper our interpretation of existing literature.

In light of these shortcomings, an approach which combines a large, age-diverse sample with DXA-derived BF may help illuminate the relationship between grip strength and BP. Accordingly, this study sought to examine the association between grip strength, BP, and odds for elevated BP in 9424 individuals aged 18–92 years while controlling for or stratifying by BMI or BF%.

## Methods

### Participant characteristics

Participants were recruited through the GenoFit study, a large dual-site, cross-sectional study that took place in Ireland between September 2017 and October 2020 that sought to explore the relationship between genetics, fitness, lifestyle and health [25]. A total of 10,546 people aged between 18–92 years participated in a single, one-hour visit, during which biological samples were gathered and a suite of phenotypic measurements was collected. The sample for the present study was refined to include 9424 individuals. Those that were excluded did not meet the following eligibility criteria: free from any severe cognitive disorder and musculoskeletal impairment/injury that may affect grip strength (hand, wrist or arm injuries, peripheral neuropathies including carpal tunnel syndrome), able/willing to have a DXA scan, and be willing and able to provide written informed consent. Ethical approval was provided by the Human Research Ethics Committee, University College Dublin. Informed consent was obtained from all subjects.

### Anthropometry

Height and body mass were measured using a SECA stadiometer and weight scales (SECA, Hamburg, Germany). Participants were dressed lightly and without footwear. Body mass index was calculated as body mass divided by height squared (kg/m^2^). Dual energy x-ray absorptiometry was used to determine level of BF, expressed as BF% (fat mass divided by overall body mass, multiplied by 100).

### Grip strength determination

Grip strength was measured using a digital hand-held dynamometer (JLW Instruments, Chicago, IL, USA) as described previously [9]. While standing, participants performed two maximal attempts with each hand (~3 s each) with their arm positioned straight by their side. The average of the highest scores from each hand was considered for the analysis [26, 27]. For a secondary analysis incorporating low grip strength, sex-specific thresholds were classified as >2SDs below the mean of those aged 20–39 years within the GenoFit cohort [<33.95 kg for males and < 21.68 kg for females (males: *n* = 1842, mean = 51.03 kg, SD = 8.54 kg; females: *n* = 1705, mean = 32.06 kg, SD = 5.19 kg)] [9].

### BP measurement and elevated BP classification

Blood pressure was measured using a digital sphygmomanometer (UA-705, A&D Company, Tokyo, Japan) following a resting period of at least five minutes. Participants were seated comfortably on a back-supporting chair, with both feet on the floor and their arms placed on an armrest so that the mid-humerus was approximately in line with the level of the heart. The cuff was placed on a bare arm approximately one inch above the cubital fossa. Participants were instructed to keep their arms relaxed, breath normally, and remain silent during the measurement. One measurement was taken, and where the first was interrupted or unusual (concerning the participants normative), a second measurement was performed. SBP and DBP values were taken directly from the sphygmomanometer, while pulse pressure (PP) was defined as the difference between SBP and DBP, and mean arterial pressure (MAP) was calculated as: DBP + 1/3(PP). Elevated BP was classified as SBP ≥ 130 mmHg and DBP ≥ 85 mmHg (Hypertension was not classified due to the number of available BP measurements, and to maximise numbers in each group for statistical analyses). Individuals consuming anti-hypertensive medication were not included in the present study.

### Covariates

Smoking status, alcohol consumption, the prevalence of diseases/disorder, level of physical activity, and educational attainment were assessed using a self-reported questionnaire [9]. Specifically, smoking status was categorised as: 1) never smoked (never smoked / smoked <100 cigarettes in lifetime), 2) previous smoker (smoked ≥100 cigarettes in lifetime but has now stopped smoking), and 3) current smoker (smoked ≥100 cigarettes in lifetime and currently smoking). Alcohol consumption, measured as the number of standard units of alcohol consumed per week, was determined by asking: “On average, how many standard drinks do you drink per week?” The presence of 56 diseases/disorders (cancers, heart diseases/disorders, skin disorders, digestive and bowel disorders, breathing disorders, bone and joint disorders, pain disorders, mental health conditions, brain/neurological disorders, diabetes) was established by asking: “Have you ever received a medical diagnosis from a doctor for any of the following conditions?” Physical activity level was determined by asking: “How many days per week do you do at least 30 min of moderate-intensity exercise that increases your breathing and heart rate (e.g. brisk walking, jogging, cycling, swimming)?” Lastly, educational attainment was assessed by asking: “What is the highest level of education you have completed to date (no formal education, primary, lower secondary, higher secondary, third level, or postgraduate)?”

### Statistical analysis

Unless stated otherwise, data are presented as means ± standard deviations (SD). Individual samples Student’s *T*-test and Chi-square tests were used to assess between-group differences for continuous and categorical variables. Pearson’s correlation coefficient was used to assess the association between grip strength, BMI/BF% and BP. The association between BP domains and grip strength was further examined using linear regression with adjustment for potentially relevant confounders, including sex, age, BMI, disease prevalence, activity levels, smoking status, education, and alcohol consumption. Analysis of covariance (ANCOVA) was used to determine differences in grip strength between those with elevated BP and those with normal BP, overall, and according to BMI and BF%. For this, categorisation into three BMI groups ( < 25 kg/m^2^, 25–29.9 kg/m^2^ and ≥30 kg/m^2^ for both sexes) and BF% tertiles (<20.3%, ≥20.3– < 27.5% and ≥27.5% for males and <29.7%, ≥29.7– < 36.9% and ≥36.9% for females) was considered appropriate to maximise the number of individuals with elevated BP in each group. Binary logistic regression was used to determine the odds ratios (ORs) for elevated BP according to grip strength stratified by BMI and BF%. Supplementary binary logistic regression models were used to assess: 1) the ORs for elevated BP according to low grip strength, stratified by BF%, and 2) the ORs for elevated BP according to grip strength in young (18–39 years, *n* = 3572), middle aged (40–59 years, *n* = 4372) and older (≥60 years, *n* = 1480) individuals, stratified by BF%. ANCOVA and binary logistic regression models were adjusted when appropriate for the aforementioned potential confounders, including sex, age, BMI, disease prevalence, activity levels, smoking status, education, and alcohol consumption. All statistical analyses were performed using the SPSS software (Version 27, IBM SPSS Inc., Chicago, Il, USA) with statistical significance set at *p* < 0.05 for all tests. Data visualisations were developed using Prism (Version 9.3.1, GraphPad, Prism, San Diego, CA, USA).

## Results

### Study sample

The characteristics of the study sample according to sex are displayed in Table 1. In total, 9424 people aged between 18–92 years participated in this study (males, *n* = 4046, mean age = 42.5 ± 13.3 years, age range = 18–92 years; and females, *n* = 5378, mean age = 46.5 ± 13.1, age range = 18–87 years).

**Table 1:** Participant characteristics according to sex.

Parameter	Total (*n* = 9424)	Males (*n* = 4046)	Females (*n* = 5378)	*p*-value
Age (years)	44.8 ± 13.4	42.5 ± 13.3	46.5 ± 13.1	<0.001
Age range (years)	18–92	18–92	18–87	
Height (cm)	170.9 ± 9.5	178.9 ± 6.7	164.9 ± 6.3	<0.001
Body mass (kg)	73.9 ± 14.1	83.6 ± 12.0	66.5 ± 10.8	<0.001
Body mass index (kg/m^2^)	25.2 ± 3.7	26.1 ± 3.3	24.5 ± 3.8	<0.001
*Body mass index, n (%)*				
Underweight	87 (1.0)	4 (0.1)	83 (1.5)	<0.001
Normal weight	4838 (51.3)	1580 (39.1)	3258 (60.6)	
Overweight	3582 (38.0)	1993 (49.3)	1589 (29.5)	
Obese	917 (9.7)	469 (11.6)	448 (8.3)	
Body fat %	29.3 ± 9.0	24 ± 7.6	33.3 ± 7.7	<0.001
Grip strength (kg)	38.5 ± 11.7	49.3 ± 8.5	30.3 ± 5.4	<0.001
Low grip strength, *n* (%)	402 (4.3)	146 (3.6)	256 (4.8)	0.006
*Blood pressure domains*				
Systolic blood pressure (mmHg)	124.4 ± 15.6	129.5 ± 13.7	120.6 ± 15.9	<0.001
Diastolic blood pressure (mmHg)	75.7 ± 10.0	76.5 ± 10.0	75.1 ± 10.0	<0.001
Pulse pressure (mmHg)	48.7 ± 12.6	53.0 ± 11.7	45.5 ± 12.2	<0.001
Mean arterial pressure (mmHg)	91.9 ± 10.6	94.1 ± 9.9	90.2 ± 10.8	<0.001
Elevated BP, *n* (%)	1112 (11.8)	598 (14.8)	514 (9.6)	<0.001
*Education, n (%)*				
No formal education	5 (0.1)	4 (0.1)	1 (<0.1)	<0.001
Primary education	68 (0.7)	33 (0.8)	35 (0.7)	
Lower secondary	319 (3.4)	167 (4.1)	152 (2.8)	
Higher secondary	1282 (13.6	558 (13.8)	724 (13.5)	
Third-level degree	5101 (54.1)	2119 (52.4)	2982 (55.4)	
Postgraduate degree	2649 (28.1)	1165 (28.8)	1484 (27.6)	
*Smoking status, n (%)*				
Never (<100 cigarettes)	5614 (59.6)	2430 (60.1)	3184 (59.2)	0.028
Previous smoker (>100 cigarettes)	1982 (21)	802 (19.8)	1180 (21.9)	
Current smoker (>100 cigarettes)	1828 (19.4)	814 (20.1)	1014 (18.9)	
Alcohol consumption (units/week)	6.6 ± 6.1	8.3 ± 7.2	5.2 ± 4.7	<0.001
Number of diseases/disorders	1.1 ± 1.2	0.9 ± 1.0	1.3 ± 1.3	<0.001
Physical activity^a^	4.1 ± 2.0	4.3 ± 2.0	4.0 ± 2.1	<0.001

^a^Days per week performing ≥30 min moderate intensity exercise.

The overall prevalence of elevated BP was 11.8% (Table 2). Elevated BP was significantly more prevalent in males than females overall (14.8% vs 9.6%, *p* < 0.001), and within each adiposity category. Elevated BP was least prevalent in females within the first BF% tertile (5.3%) and most prevalent in males with a BMI ≥ 30 kg/m^2^ (29.4%) (Table 2).

**Table 2:** Prevalence of elevated blood pressure (BP) according to body mass index (BMI) and body fat (BF) %.

	Total (*n* = 9424)	Males (*n* = 4046)	Females (*n* = 5378)	χ^2^ test
*Full sample (n* = *9424)*				
Normal BP	8312 (88.2)	3448 (85.2)	4864 (90.4)	<0.001
Elevated BP	1112 (11.8)	598 (14.8)	514 (9.6)	
*BMI* < *25* *kg/m*^*2*^ *(n* = *4925)*				
Normal BP	4581 (93.0)	1449 (91.5)	3132 (93.7)	0.002
Elevated BP	344 (7.0)	135 (8.5)	209 (6.3)	
*BMI 25–29.9* *kg/m*^*2*^ *(n* = *3582)*				
Normal BP	3054 (85.3)	1668 (83.7)	1386 (87.2)	0.003
Elevated BP	528 (14.7)	325 (16.3)	203 (12.8)	
*BMI* ≥ *30* *kg/m*^*2*^ *(n* = *917)*				
Normal BP	677 (73.8)	331 (70.6)	346 (77.2)	0.022
Elevated BP	240 (26.2)	138 (29.4)	102 (22.8)	
*BF% tertile 1 (n* = *3113)*				
Normal BP	2915 (93.6)	1234 (92.2)	1681 (94.7)	0.005
Elevated BP	198 (6.4)	104 (7.8)	94 (5.3)	
*BF% tertile 2 (n* = *3151)*				
Normal BP	2817 (89.4)	1157 (86.2)	1660 (91.8)	<0.001
Elevated BP	334 (10.6)	186 (13.8)	148 (8.2)	
*BF% tertile 3 (n* = *3160)*				
Normal BP	2580 (81.6)	1057 (77.4)	1523 (84.8)	<0.001
Elevated BP	580 (18.4)	308 (22.6)	272 (15.2)	

Data displayed as *n* (%); BF% tertiles = 1: <20.3%, 2: ≥20.3– < 27.5% and 3: ≥27.5% for males and 1: <29.7%, 2: ≥29.7– < 36.9% and 3: ≥36.9% for females.

### Associations between grip strength, BMI/BF% and BP domains

**Fig. 1:**
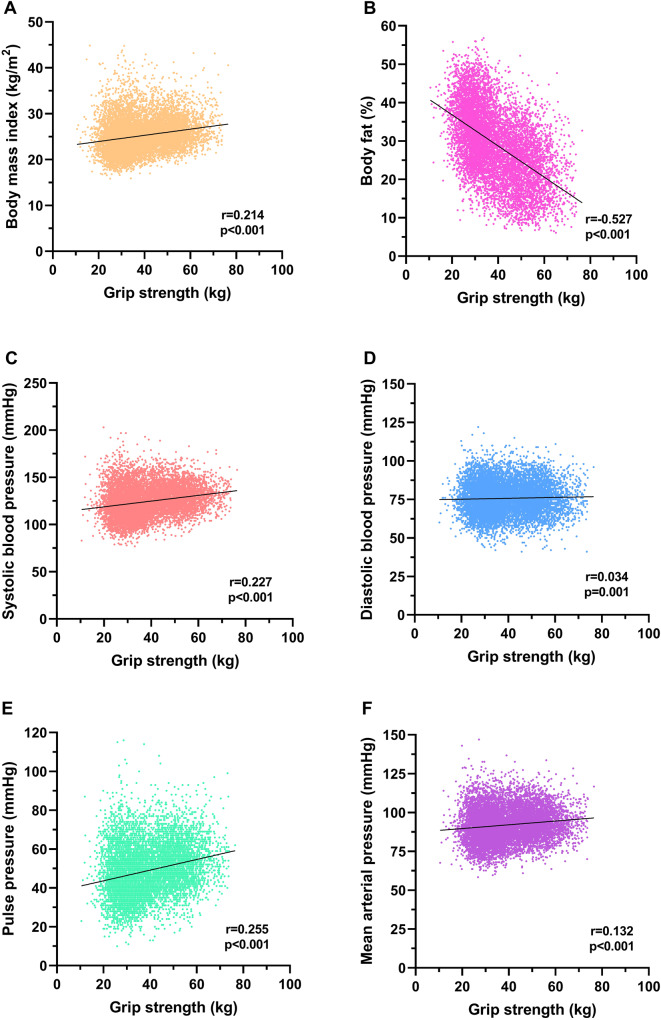
Associations between grip strength, measures of adiposity and blood pressure domains. **A** grip strength and body mass index, **B** grip strength and body fat % and **C**–**F** grip strength and blood pressure domains.

Grip strength was positively associated with BMI (*r* = 0.214, *p* < 0.001) and negatively associated with BF% (*r* = −0.527, *p* < 0.001) (Fig. 1). Grip strength was positively associated with SBP (*r* = 0.227, *p* < 0.001), DBP (*r* = 0.034, *p* = 0.001), MAP (*r* = 0.132, *p* < 0.001) and PP (*r* = 0.255, *p* < 0.001) (Fig. 1).

Overall, SBP, PP, and MAP were positively associated with grip strength (all *p* < 0.001) after controlling for sex, age, BMI, disease prevalence, activity level, smoking status, education, and alcohol consumption (Table 3). No significant association was observed between DBP and grip strength (*p* = 0.786). Using the same regression model but substituting BF% for BMI, SBP, DBP, PP and MAP were all positively associated with grip strength (all *p* < 0.001) (Table 3).

**Table 3:** Association between blood pressure domains and grip strength.

	Grip strength (kg)			
Blood pressure domain	β	95% CI	*p*-value	R^2^
DBP (mmHg)^a^	0.002	0.012–0.016	0.786	0.693
SBP (mmHg)^a^	0.033	0.024–0.042	<0.001	0.695
MAP (mmHg)^a^	0.024	0.011–0.037	<0.001	0.694
PP (mmHg)^a^	0.046	0.035–0.057	<0.001	0.695
DBP (mmHg)^b^	0.044	0.030–0.057	<0.001	0.692
SBP (mmHg)^b^	0.052	0.043–0.062	<0.001	0.695
MAP (mmHg)^b^	0.064	0.050–0.077	<0.001	0.694
PP (mmHg)^b^	0.049	0.038–0.060	<0.001	0.693

^a^Adjusted for sex, age, BMI, disease prevalence, activity levels, smoking status, education and alcohol consumption.

^b^Model ^a^ except body fat % replaces BMI; *DBP* diastolic blood pressure, *SBP* systolic blood pressure, *MAP* mean arterial pressure, *PP* pulse pressure, *n* = 9424.

### Differences in grip strength between those with normal BP and those with elevated BP according to BMI or BF%

Overall, those with elevated BP had significantly higher grip strength compared to those with normal BP (39.17 kg vs 38.38 kg, *p* < 0.001), while controlling for several confounders (Table 4). When stratified by BMI, those with elevated BP and a BMI of 25–29.9 kg/m^2^ or ≥30 kg/m^2^ had significantly higher grip strength than those with normal BP in the corresponding BMI category (42.08 kg vs 41.10 kg, *p* = 0.003 and 41.34 kg vs 40.03 kg, *p* = 0.033, respectively), whereas no significant differences were observed between individuals with elevated BP and normal BP with a BMI < 25 kg/m^2^. When stratified by BF%, those with elevated BP in the highest BF% tertile had significantly greater grip strength than those with normal BP in the same tertile (37.95 kg vs 36.52 kg, *p* < 0.001). No significant differences were observed between individuals with elevated BP and individuals with normal BP in BF% tertiles 1 and 2 (Table 4).

**Table 4:** Differences in grip strength between those with normal blood pressure (BP) and those with elevated BP according to body mass index (BMI) or body fat (BF) %.

	Normal BP (*n* = 8312)	Elevated BP (*n* = 1112)	% difference	*p*-value
BMI/BF% category	Grip strength (kg)			
*Full sample (n* = *9424)*				
Total^a^	38.38 (0.07)	39.17 (0.20)	+2.1%	<0.001
Males^b^	49.13 (0.14)	50.21 (0.34)	+2.2%	0.003
Females^b^	30.28 (0.07)	30.80 (0.22)	+1.7%	0.026
*BMI* < *25* *kg/m*^*2*^ *(n* = *4925)*				
Total^c^	36.22 (0.09)	36.02 (0.32)	–0.6%	0.549
Males^d^	48.24 (0.21)	47.48 (0.69)	–1.6%	0.289
Females^d^	30.35 (0.09)	30.54 (0.34)	+0.6%	0.573
*BMI 25–29.9* *kg/m*^*2*^*(n* = *3582)*				
Total^c^	41.10 (0.13)	42.08 (0.30)	+2.4%	0.003
Males^d^	49.87 (0.19)	51.07 (0.44)	+2.4%	0.014
Females^d^	29.89 (0.12)	30.65 (0.32)	+2.5%	0.028
*BMI* ≥ *30* *kg/m*^*2*^*(n* = *917)*				
Total^c^	40.03 (0.31)	41.34 (0.52)	+3.3%	0.033
Males^d^	49.49 (0.47)	50.46 (0.73)	+2.0%	0.268
Females^d^	29.55 (0.27)	30.74 (0.51)	+4.0%	0.040
*BF% tertile 1 (n* = *3113)*				
Total^c^	40.24 (0.12)	40.58 (0.47)	+0.8%	0.490
Males^d^	51.33 (0.24)	51.34 (0.82)	+0.1%	0.991
Females^d^	31.89 (0.12)	32.54 (0.52)	+2.0%	0.216
*BF% tertile 2 (n* = *3151)*				
Total^c^	38.29 (0.12)	38.86 (0.36)	+1.5%	0.132
Males^d^	49.28 (0.24)	50.35 (0.61)	+2.2%	0.104
Females^d^	30.17 (0.12)	30.58 (0.40)	+1.4%	0.333
*BF% tertile 3 (n* = *3160)*				
Total^c^	36.52 (0.13)	37.95 (0.27)	+3.9%	<0.001
Males^d^	46.71 (0.24)	48.70 (0.45)	+4.3%	<0.001
Females^d^	28.76 (0.13)	29.61 (0.30)	+3.0%	0.009

^a^Adjusted for sex, age, BMI, disease prevalence, activity levels, smoking status, education and alcohol consumption.

^b^Model ^a^ without sex.

^c^Model ^a^ without BMI.

^d^Model ^a^ without BMI and sex; data presented as mean (standard error of mean).

Overall, females with elevated BP had significantly higher grip strength than those with normal BP (30.80 kg vs 30.28 kg, *p* = 0.026). Females with elevated BP had significantly higher grip strength than those with normal BP in the 25–29.9 kg/m^2^ and ≥30 kg/m^2^ BMI categories (30.65 kg vs 29.89 kg, *p* = 0.028 and 30.74 kg vs 29.55 kg, *p* = 0.040, respectively), but not in the <25 kg/m^2^ group. Similarly, females with elevated BP had significantly greater grip strength than those with normal BP in BF% tertile 3 (29.61 kg vs 28.76 kg, *p* = 0.009), while no significant differences were observed in BF% tertiles 1 and 2 (Table 4).

Overall, males with elevated BP had significantly higher grip strength than those with normal BP (50.21 kg vs 49.13 kg, *p* = 0.003). In the 25–29.9 kg/m^2^ BMI category and third BF% tertile, males with elevated BP had significantly higher grip strength compared to males with normal BP (51.07 kg vs 49.87 kg, *p* = 0.014 and 48.70 kg vs 46.71 kg, *p* < 0.001, respectively). No significant differences in grip strength were observed between males with elevated BP and normal BP in the <25 kg/m^2^ and ≥30 kg/m^2^ BMI groups, and in BF% tertiles 1 and 2 (Table 4).

### Odds for elevated BP according to grip strength and low grip strength, stratified by BMI or BF%

Overall, higher grip strength was associated with an increased odds for elevated BP (OR = 1.014, 95% CI = 1.004–1.024, *p* = 0.004), after controlling for sex, age, BMI, disease prevalence, activity level, smoking status, education and alcohol consumption (Table 5). Following stratification by BMI/BF% and controlling for the same confounders (except BMI), positive associations between grip strength and elevated BP odds were observed in individuals within the 25–29.9 kg/m^2^ and ≥30 kg/m^2^ BMI groups (OR = 1.025, 95% CI = 1.010–1.039, *p* < 0.001 and OR = 1.018, 95% CI = 1.004–1.031, *p* = 0.010, respectively) and BF% tertile 3 (OR = 1.036, 95% CI = 1.022–1.051, *p* < 0.001). No significant differences were observed for those with a BMI < 25 kg/m^2^, or BF% within tertiles 1 or 2 (Table 5).

**Table 5:** Odds for elevated blood pressure according to grip strength, stratified by body mass index (BMI) or body fat (BF) %.

	β	SE	OR (95% CI)	*p*-value
*Full sample (n* = *9424)*				
Total^a^	0.014	0.005	1.014 (1.004–1.024)	0.004
Males^b^	0.012	0.009	1.012 (1.000–1.023)	0.042
Females^b^	0.027	0.010	1.027 (1.008–1.047)	0.005
*BMI* < *25* *kg/m*^*2*^ *(n* = *4925)*				
Total^c^	–0.001	0.009	0.999 (0.981–1.017)	0.876
Males^d^	–0.010	0.012	0.990 (0.967–1.013)	0.377
Females^d^	0.016	0.015	1.016 (0.986–1.047)	0.307
*BMI 25–29.9* *kg/m*^*2*^ *(n* = *3582)*				
Total^c^	0.024	0.007	1.025 (1.010–1.039)	<0.001
Males^d^	0.022	0.008	1.023 (1.007–1.039)	0.005
Females^d^	0.036	0.016	1.037 (1.005–1.070)	0.024
*BMI* ≥ *30* *kg/m*^*2*^ *(n* = *917)*				
Total^c^	0.018	0.007	1.018 (1.004–1.031)	0.010
Males^d^	0.014	0.013	1.015 (0.990–1.040)	0.250
Females^d^	0.049	0.023	1.050 (1.004–1.098)	0.034
*BF% tertile 1 (n* = *3113)*				
Total^c^	0.012	0.011	1.012 (0.990–1.034)	0.293
Males^d^	0.008	0.021	1.008 (0.968–1.050)	0.697
Females^d^	0.036	0.022	1.037 (0.994–1.082)	0.092
*BF% tertile 2 (n* = *3151)*				
Total^c^	0.017	0.009	1.017 (1.000–1.035)	0.055
Males^d^	0.019	0.010	1.019 (0.999–1.039)	0.062
Females^d^	0.024	0.018	1.024 (0.988–1.062)	0.190
*BF% tertile 3 (n* = *3160)*				
Total^c^	0.035	0.007	1.036 (1.022–1.051)	<0.001
Males^d^	0.034	0.009	1.035 (1.018–1.053)	<0.001
Females^d^	0.037	0.013	1.038 (1.011–1.065)	0.006

^a^Adjusted for sex, age, BMI, disease prevalence, activity levels, smoking status, education and alcohol consumption.

^b^Model ^a^ without sex.

^c^Model ^a^ without BMI.

^d^Model ^a^ without BMI and sex.

In females, higher grip strength was associated with increased odds for elevated BP across the full sample (OR = 1.027, 95% CI = 1.008–1.047, *p* = 0.005), and in those with a BMI of 25–29.9 kg/m^2^ or ≥30 kg/m^2^ (OR = 1.037, 95% CI = 1.005–1.070, *p* = 0.024 and OR = 1.050, 95% CI = 1.004–1.098, *p* = 0.034, respectively), or BF% within tertile 3 (OR = 1.038, 95% CI = 1.011–1.065, *p* = 0.006), but not in those with a BMI < 25 kg/m^2^ or BF% within tertiles 1 or 2. In males, higher grip strength was associated with an increased odds for elevated BP overall (OR = 1.012, 95% CI = 1.000–1.023, *p* = 0.042), and in those with a BMI of 25–29.9 kg/m^2^ (OR = 1.023, 95% CI = 1.007–1.039, *p* = 0.005), or BF% within tertile 3 (OR = 1.035, 95% CI = 1.018–1.053, *p* < 0.001), but not in those with a BMI < 25 kg/m^2^ or ≥30 kg/m^2^, or BF% within tertiles 1 or 2 (Table 5).

Those with low grip strength and a BF% within the highest tertile had significantly lower odds for elevated BP (OR = 0.514, 95% CI = 0.341–0.775, *p* = 0.002) (Table 6). The association remained significant when stratified by sex (males: OR = 0.387, 95% CI = 0.199–0.754, *p* = 0.005; females: OR = 0.573, 95% CI = 0.335–0.981, *p* = 0.043). In contrast, those with low grip strength and a BF% within the lowest tertile had significantly higher odds for elevated blood pressure (OR = 2.162, 95% CI = 1.026–4.555, *p* = 0.043), although the ORs were not significant when stratified by sex. No significant associations were found within BF% tertile 2.

**Table 6:** Odds for elevated blood pressure according to the presence of low grip strength, stratified by body fat (BF) %.

	b	SE	OR (95% CI)	*p*-value
*BF% tertile 1 (n* = *3113)*				
Total^a^	0.771	0.380	2.162 (1.026–4.555)	0.043
Males^b^	0.874	0.562	2.396 (0.797–7.205)	0.120
Females^b^	0.765	0.486	2.150 (0.829–5.572)	0.115
*BF% tertile 2 (n* = *3151)*				
Total^a^	−0.277	0.297	0.758 (0.424–1.357)	0.351
Males^b^	−0.309	0.442	0.734 (0.309–1.746)	0.485
Females^b^	−0.263	0.401	0.769 (0.350–1.685)	0.511
*BF% tertile 3 (n* = *3160)*				
Total^a^	−0.665	0.210	0.514 (0.341–0.775)	0.002
Males^b^	−0.949	0.340	0.387 (0.199–0.754)	0.005
Females^b^	−0.557	0.274	0.573 (0.335–0.981)	0.043

^a^Adjusted for sex, age, disease prevalence, activity levels, smoking status, education and alcohol consumption.

^b^Model ^a^ without sex.

### Odds for elevated BP according to grip strength in young, middle-aged and older individuals, stratified by BF %

Within the highest BF% tertile, higher grip strength was associated with significantly greater odds for elevated BP in young and middle-aged individuals (OR = 1.065, 95% CI = 1.031–1.100, *p* < 0.001 and OR = 1.024, 95% CI = 1.005–1.043, *p* = 0.015, respectively), but not older individuals (Table 7). No significant associations were observed in BF% tertiles 1 or 2.

**Table 7:** Odds for elevated blood pressure according to grip strength in young, middle-aged and older individuals, stratified by body fat (BF) %.

	β	SE	OR (95% CI)	*p*-value
*BF% tertile 1 (n* = *3113)*				
Young	0.017	0.017	1.017 (0.984–1.050)	0.317
Middle-aged	0.007	0.017	1.007 (0.975–1.041)	0.660
Older	−0.023	0.036	0.977 (0.911–1.048)	0.516
*BF% tertile 2 (n* = *3151)*				
Young	0.001	0.017	1.001 (0.969–1.034)	0.957
Middle-aged	0.012	0.012	1.012 (0.988–1.036)	0.332
Older	0.035	0.021	1.036 (0.994–1.078)	0.091
*BF% tertile 3 (n* = *3160)*				
Young	0.063	0.017	1.065 (1.031–1.100)	<0.001
Middle-aged	0.023	0.010	1.024 (1.005–1.043)	0.015
Older	0.019	0.014	1.019 (0.990–1.048)	0.195

All models adjusted for sex, disease prevalence, activity levels, smoking status, education and alcohol consumption; young = 18–39 years, middle aged = 40–59 years, older = ≥60 years.

## Discussion

The principal findings of this study are: 1) grip strength was positively related to SBP, PP, MAP, and DBP (DBP association was dependent on the measure of adiposity included in the model); 2) overall, those with elevated BP had significantly greater grip strength than those with normal BP. However, following stratification for BMI or BF% this was only observed in overweight or obese individuals, and subjects within the highest BF% tertile; 3) similarly, although higher grip strength was generally associated with an increased odds for elevated BP, after stratification the increased odds was only observed in overweight or obese individuals, and those within the highest BF% tertile; 4) those with low grip strength and a BF% in the highest tertile had significantly lower odds for elevated BP, whereas those with low grip strength and a BF% within the lowest tertile had significantly higher odds for elevated BP; 5) adiposity appears to be a particularly relevant mediator of the relationship between BP and muscular strength in early adulthood.

Collectively, our findings support the potential relevance of adiposity in mediating the relationship between muscular fitness and BP. Although we found grip strength to be positively related to BP and odds for elevated BP in overweight and obese individuals, we observed a negative, although non-significant association in those with a BMI < 25 kg/m^2^ (Tables 4 and 5). The mediating effect of adiposity was particularly pronounced following stratification according to DXA derived BF%, where only those in the highest BF% tertile displayed positive associations between grip strength and elevated BP (Tables 4 and 5). Although it is noteworthy that the ORs reported in Table 5 are relatively small, it is important to consider that grip strength was included as a continuous variable and so, the ORs reflect a 1 kg change in grip strength. With this in mind, the clinical relevance of our findings may become more apparent in scenarios where grip strength classifications are used (e.g. low grip strength vs normal grip strength). Notably, in a secondary analysis using this approach, we found considerably larger ORs, indicating lower odds for elevated BP in those with low grip strength and a high BF%, and higher odds for elevated BP in those with low grip strength and low BF% (Table 6). Collectively, our findings suggest that grip strength may be positively associated with odds for elevated BP in overweight and obese individuals or those with a high BF%. However, having a BMI < 25 kg/m^2^ or lower BF% (in this case <27.5% for males or <36.9% for females) may neutralise, or even reverse this association. While the precise mechanisms underpinning these observations have not been fully elucidated, there are several plausible explanations.

As discussed, it is well established that muscular fitness has strong positive effects on cardiovascular health, and on the other hand, increasing adiposity has powerful deleterious effects. Interestingly, recent data suggest that high levels of adiposity negate the protective benefits of muscular fitness on cardiovascular health [28, 29]. Indeed, using the ‘fit-but-fat’ paradigm, studies have reported that even if an individual has high muscular fitness, having a high BMI or BF% dramatically inhibits the benefits of muscular fitness to a point comparable with the cardiovascular risk associated with being ‘unfit-and-fat’ [28, 29]. These studies are supported by others demonstrating high levels of physical activity or cardiorespiratory fitness are not enough to outweigh the negative consequences of being overweight or obese [30, 31]. With this in mind, it is plausible that our findings are similarly evoked by the deleterious effects that adiposity (specifically a BMI ≥ 25 kg/m^2^, or a BF ≥ 27.5% for males or ≥36.9% for females) produces on the usual protective effects of muscular fitness. Nevertheless, other data suggest high muscular fitness may somewhat compensate for the negative consequences of adiposity on cardiometabolic health [32], thus, more studies are needed to clarify the extent of interplay between adiposity, muscular fitness and cardiovascular health.

Secondly, and somewhat relatedly, methods used to control for adiposity have been inconsistent, with some studies electing to control for BMI as a continuous variable [15, 17], and others choosing to stratify by BMI according to pre-defined categories [18, 19]. In this scenario, including BMI as a continuous variable assumes that the associated risk is linear across BMI values, which may not be true. Indeed, many studies have reported non-linear associations between BMI, BP and grip strength [33–35], and so, adjusting for BMI as a continuous variable may risk inaccurately generalising associations across BMI strata. Interestingly, while the linear regressions in this study suggest positive associations between BP and grip strength (Table 3), stratification by BMI or BF% revealed nuanced and potentially more accurate associations according to adiposity (Tables 4 and 5). The impact of using different adjustment methods has also been highlighted in recent studies where generally positive associations between BP and grip strength were observed after linear regression, while upon stratification these associations remained present among overweight and obese individuals, but were absent among normal weight individuals [18, 19]. Evidently, adiposity is a physiologically mediating factor that warrants consideration, although the choice of statistical analysis is of similar importance and should be considered when interpreting study findings.

Thirdly, obesity-related architectural and functional changes to the peripheral vasculature may help explain the findings of our study. For example, obesity induces endothelial dysfunction, which alters vascular tone, leading to increased peripheral vascular resistance and elevated BP [36, 37]. On the other hand, individuals with increased peripheral vascular resistance may have greater potential to produce maximal grip strength due to the concomitant presence of heightened sympathetic tone that may facilitate greater muscle activation [38, 39]. Notably, such sympathetic overactivity is most prevalent among overweight and obese individuals [40]. Therefore, it is plausible that obesity-related differences in the peripheral vasculature and sympathetic activity evoke a positive relationship between grip strength and BP. In contrast, the absence of such alterations in individuals of lower adiposity may help to uncover a potentially therapeutic relationship between skeletal muscle health and cardiovascular health, although more data are needed to confirm this.

Interestingly, we observed several nuances in the association between BP and grip strength. Firstly, the mediating relevance of adiposity on the relationship between BP and muscular strength is most notable in early and middle adulthood, rather than late adulthood (Table 7). This suggests that the regulation of BP may be particularly complex in older individuals, and that other factors may influence the relationship between BP and grip strength, rather than adiposity. In this respect, the association between BP and measures of adiposity has been shown to be weaker in older subjects compared to younger subjects, which may somewhat help to explain our findings [41]. Secondly, the associations were stronger among females, compared to males (Tables 4 and 5). In this regard, evidence relating to the effect of sex on the association between BP and grip strength is conflicting. For example, although studies have reported similar sex-specific differences to those observed in our study [42, 43], some have reported stronger associations among males [18, 19], and another observed significant associations in both sexes [17]. Interestingly in the present study, the sex-specific differences were most notable when BMI was considered as the measure of adiposity, rather than DXA-derived figures. Given that the correlation between BMI and BF% differs according to sex [44], it is plausible that the nuances were evoked by the tendency of BMI to overestimate adiposity in males compared to females [24, 45]. This phenomenon may help explain the weaker associations between grip strength and BP in males when stratifying by BMI, and ultimately underpins the benefits of using DXA for illuminating the true association between muscle strength, BP, and adiposity. Thirdly, while we observed significant associations between SBP, MAP, PP and DBP, the association with DBP was dependent on the measure of adiposity included in the regression model (Table 3). Similar to sex-specific data, data surrounding the relationship between grip strength and BP domains are also inconsistent. Indeed, while one study reported findings consistent with ours [20], another reported associations with DBP but not SBP [42], and a further study reported associations with SBP and DBP [18]. It is clear, therefore, that further research is needed to confirm: a) the mediating relevance of sex on the association between grip strength and BP; and b) the relationship between grip strength and BP domains (SBP, DBP, PP and MAP).

There are several strengths and limitations to this study that should be noted. The main strengths include the large, comprehensively phenotyped study sample and the incorporation of DXA-derived BF%. The principal limitation is the cross-sectional design, which ultimately prevents the inference of causal relationship between BP and grip strength. Secondly, while adaptation of the GenoFit study protocol was not possible in this instance, measuring BP two or three times rather than once would have been beneficial. Nonetheless, the accuracy of machine coupled with strict standardisation of protocol and environment helped ensure the quality of BP readings. Thirdly, although statistically significant, the differences in grip strength observed between those with elevated BP and those with normal BP according to adiposity classification were relatively small (up to 4.3%; Table 4). Therefore, future studies are needed to determine the extent that grip strength may differ between those with normal and elevated BP in relevant adiposity categories. Finally, while categorising individuals according to adiposity is common in clinical health settings, it is worth noting that doing so leads to an inherent loss of information, whereby individuals within each category are considered somewhat homogenous. Nevertheless, in this scenario we believe categorisation to be a better suited approach for controlling for adiposity, compared to linear adjustment.

Collectively, our findings suggest higher grip strength is associated with higher BP, but only in overweight or obese individuals, or those with a high BF%. Having a BMI < 25 kg/m^2^ or lower BF% (in this instance <27.5% for males or <36.9% for females) appears to neutralise the association. The associations between BP and grip strength were stronger among females compared to males, although the differences were less apparent when DXA-derived BF was considered. More studies are needed to elucidate the underlying mechanisms by which adiposity and sex mediate the relationship between BP and muscular fitness.

## Summary

### What is known about the topic

- Strong positive correlations exist between grip strength and cardiovascular health.
- The association between grip strength and blood pressure is less clear, with positive and negative associations reported to date.
- Emerging data suggest adiposity may mediate the association, however existing studies have used BMI rather than a more accurate adiposity measure such as DXA.

### What this study adds

- Large, well-characterised study sample incorporating DXA derived body fat %.
- Showcases that adiposity mediates the association between grip strength and blood pressure and demonstrates the importance of employing appropriate statistical analyses.
- Higher grip strength is associated with higher blood pressure, but only in cases of high adiposity (BMI ≥ 25 kg/m^2^ or body fat ≥27.5% for males or ≥36.9% for females).

## Data Availability

Data may be made available upon reasonable request to the corresponding author.
